# Natural Disasters and the Dengue Epidemic During the COVID-19 Outbreak: Deadly Combination for Public Health Threats in Bangladesh

**DOI:** 10.1017/dmp.2020.493

**Published:** 2020-12-22

**Authors:** Rumana Sultana, Md. Shafiul Alam

**Affiliations:** 1 Center for Sustainable Development, University of Liberal Arts Bangladesh (ULAB), Dhanmondi, Dhaka, Bangladesh; 2 Department of Geography and Environmental Studies, University of Rajshahi, Rajshahi, Bangladesh

**Keywords:** COVID-19, Natural Disaster, Dengue, Public Health

**Figure 1.**
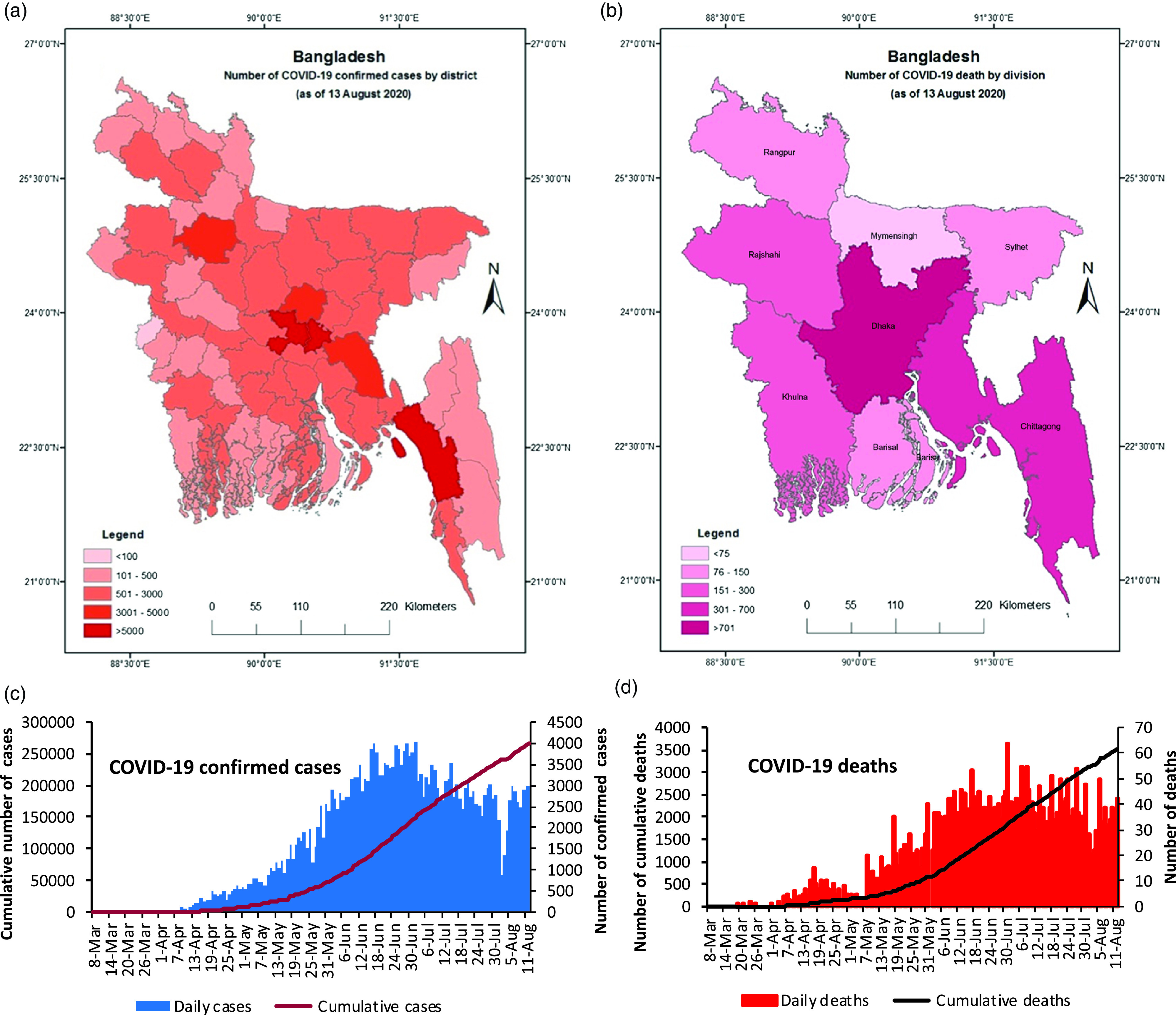
COVID-19 confirmed cases, deaths, and distribution as of August 13, 2020. (a) Number of confirmed cases with distribution by district. (b) Number of deaths with distribution by division. (c) Daily number of confirmed COVID-19 cases. (d) Daily number of COVID-19 deaths.

The outbreak of the 2019 coronavirus disease (COVID-19) impacted the world’s public health and economy tremendously, including Bangladesh. The first confirmed cases were reported in Bangladesh on March 8, 2020, and as of August 26, 2020, the total number of positive COVID-19 cases were 302 147, with 4082 dead (Figure 1). The extremely diverse cases were between 21 and 40 years of age (55%), while those over 60 years of age (48%) were fatal, where the average fatality rate was only 1.33%.^1^ As a low-income, developing, densely populated country, Bangladesh is at a higher risk of the pandemic due to fragile health systems, poor socioeconomic conditions, frequent natural disasters, an increasing number of older adults, migrants, refugees, dense cities, and other infectious diseases. According to the Directorate General of Health Services (DGHS) Bangladesh, as of August 13, 2020, only 564 intensive care unit (ICU) beds have been available in the government hospitals, and 992 physicians, 825 nurses, 572 supporting staffs, other (not elsewhere classified) 263 staffs, 212 medical technologists, 163 field staffs, 6 dental surgeons, and 2 ayurvedic specialists have been working for COVID-19 health services in the whole country.^2,3^ The number of COVID-19 medical team members and ICU beds are inadequate to support over 160 million people. To combat this pandemic, the government has decided to recruit more than 2000 physicians and 4000 nurses to the COVID-19 medical team.

Besides religious and cultural faith, personal hygiene practice, attitude toward contagious disease, misinformation, and pre-existing comorbidities make people more vulnerable to the COVID-19 pandemic. In April 2020, more than 100 000 people attended the funeral rites of a senior leader of the Islamist party, violating government lockdown rules without maintaining social distancing.^4^ Throughout the country, people are going outside with no essential need, even in the lockdown areas during the pandemic. The importance of following general guidelines of non-pharmaceutical interventions (frequent hand washing, mask use, staying at home, social distancing) has been a challenging concept for convincing many vulnerable social groups (ie, older adults, pregnant women). Also, the knowledge about COVID-19 cannot be fully understood by older adults, particularly women, due to their limited exposures to mass media and lack of awareness of activities that may make them more vulnerable to the pandemic.^5^ Moreover, natural disasters and dengue add fuel to the fire to exacerbate the risks of the COVID-19 pandemic.

Bangladesh is a disaster-prone deltaic country. As a developing country, the current challenge is how to deal with the COVID-19 pandemic in response to natural disasters and other infectious diseases. Natural disasters may have a rapid onset, extensive effects, and generate several factors that function synergistically to increase the risk of morbidity and mortality resulting from infectious diseases.^6^ Currently, Bangladesh is facing a catastrophic monsoon flood. One-third of Bangladesh is underwater, which has affected a total of over 5 million people.^7^ This year, floods are happening while Bangladesh is recovering from Cyclone Amphan and working hard to control the spread of COVID-19. Over 2 million people were evacuated during cyclone and flood disasters to the shelters, where social distancing was more difficult to achieve and enforce due to the crowded environment.

Moreover, salty water is entering the coastal community through the broken dam owing to unexpected tidal height and heavy precipitation since the third week of August 2020. This situation may increase the risk of rapid spread of COVID-19 among vulnerable social groups. The consequence of these disasters without the COVID-19 will be felt with very little life and property damage. On the other hand, natural disasters amid COVID-19 would have a more significant adverse effect on the country’s livelihoods, properties, products, services, and food security.

Between May and September, Bangladesh usually faces the highest degree of susceptibility to infectious disease outbreaks, such as dengue, chikungunya, and malaria. Over 100 000 people were diagnosed with dengue fever last year that took some 200 lives.^8^ All attention of the people in the health care sector is currently concentrated on handling the COVID-19 emergency. Due to fever being a common symptom of both COVID-19 and dengue, people’s focus also goes on only COVID-19. In this situation, if dengue continues to affect people amid COVID-19 during this monsoon, the current health crisis will escalate further and create other health emergencies.

Natural disasters and other infectious diseases, including dengue, may result in excess mortality, social crisis, poverty, lack of food security, and increased stress on limited health care. Combining preparatory and mitigation measures in the long and short term should be implemented on an emergency basis to save the vulnerable social groups from the adverse effect of cyclone, flood, and dengue amid the COVID-19 pandemic. This includes the renovation of the broken dams, effective early warning message dissemination, initiation of separate geriatrics units at public and private hospitals, and a combined natural and biological disaster preparedness program. Also, isolation shelters and dedicated ambulance and boat services should be arranged in disaster-prone areas to transport suspected COVID-19 or dengue patients to the nearest health care centers during the pandemic and catastrophe. Furthermore, policy-makers and stakeholders should promptly prepare for the management of post-COVID-19 adverse effects on vulnerable people.
